
JOURNAL INFORMATION
==============================
NLM Title Abbreviation: J Appl Clin Med Phys
Journal ID: J Appl Clin Med Phys
NLM Journal ID: 101089176
Journal ID: ACM2

Journal of Applied Clinical Medical Physics

EISSN: 1526-9914
Publisher: Wiley

ARTICLE INFORMATION
==============================
PMCID: PMC9332877
DOI: 10.1002/acm2.12934
Article ID: ACM212934
Article version: 1
Subjects: Issue Information, Issue Information

Issue Information

Electronic publication date: 2021 Nov 17
Publication date: 2021 Nov
Volume: 22
Issue: 11
First page: 1
Last page: 3
Copyright: © 2021 The Authors. Journal of Applied Clinical Medical Physics published by Wiley Periodicals, LLC on behalf of The American Association of Physicists in Medicine
License: This is an open access article under the terms of the http://creativecommons.org/licenses/by/4.0/ License, which permits use, distribution and reproduction in any medium, provided the original work is properly cited.
License URL: https://creativecommons.org/licenses/by/4.0/

Figure count: 0
Table count: 0
Page count: 3

source-schema-version-number: 2.0
cover-date: November 2021
details-of-publishers-convertor: Converter:WILEY_ML3GV2_TO_JATSPMC version:6.1.7 mode:remove_FC converted:28.07.2022

==============================

November 2021 | Volume 22, Issue 11

### JOURNAL OF APPLIED CLINICAL MEDICAL PHYSICS

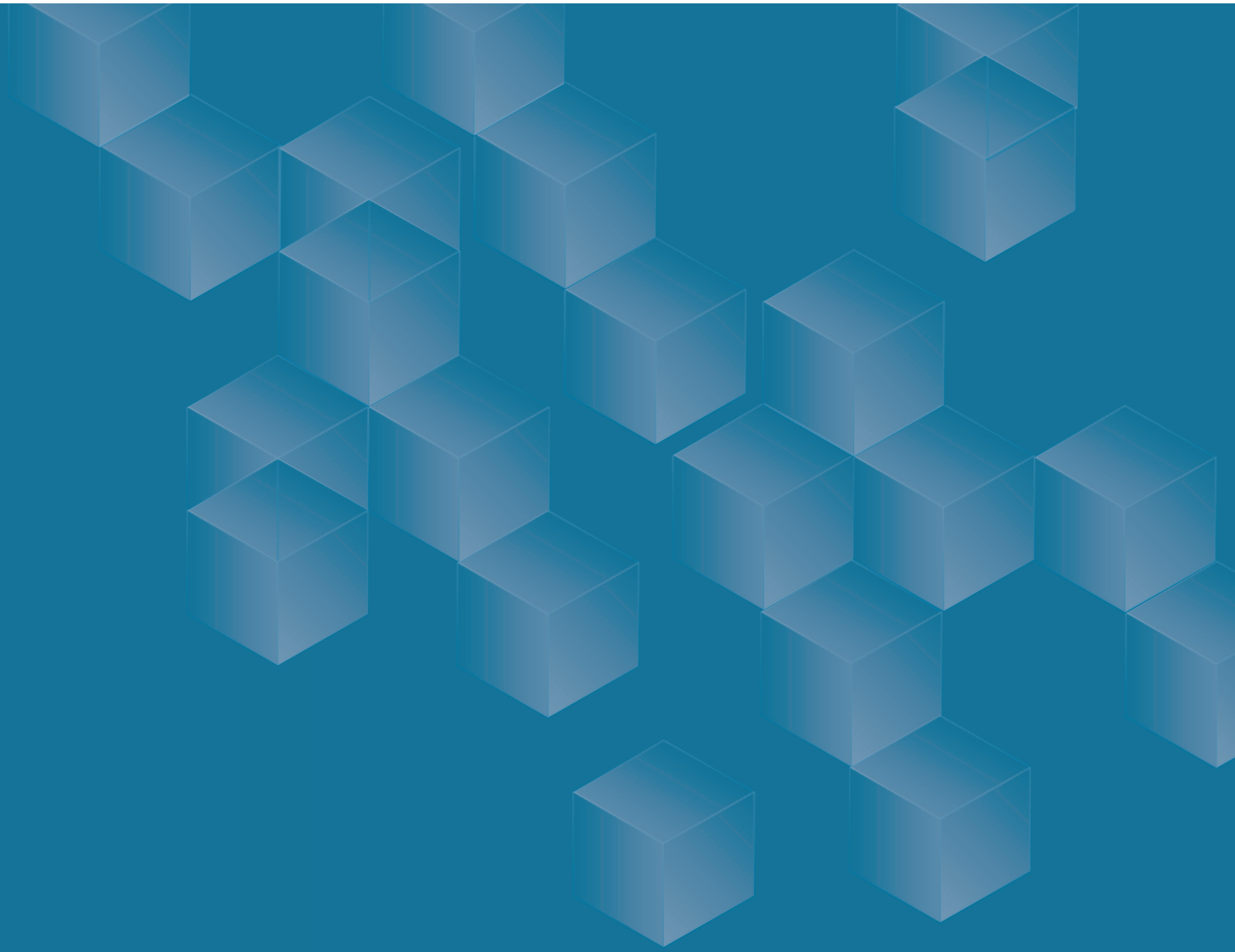

The *Journal of Applied Clinical Medical Physics* is an official journal of the AAPM, the International Organization for Medical Physics (IOMP), and the Canadian Organization of Medical Physicists (COMP).

AMERICAN ASSOCIATION of PHYSICISTS IN MEDICINE

WILEY

### Editorial Team

### Editor-in-Chief

Michael D Mills, University of Louisville Brown Cancer Center, Radiation Oncology Suite 400, 529 South Jackson Street, Louisville, KY 40202, United States

### Deputy Editors–in–Chief

Per H Halvorsen, Lahey Health and Medical Center, Burlington, MA, United States
Timothy D Solberg, University of Washington, Seattle, WA, United States

### Associate Editors—Radiation Oncology Physics

Hossein Afsharpour, *Trillium Health Partners, Mississauga, ON, Canada*
Nzhde Agazaryan, *UCLA School of Medicine, Los Angeles, CA, United States*
Munir Ahmad, *University College Lahore, Lahore, Pakistan*
Salahuddin Ahmad, *University of Oklahoma HSC, OK, United States*
Nathan Anderson, *Hospital of the University of Pennsylvania, Philadelphia, PA, United States*
Bulent Aydogan, *University of Chicago, Chicago, IL, United States*
Daniel Bailey, *Northside Hospital Cancer Institute, Atlanta, GA, United States*
Charles Bloch, *University of Washington, Seattle, WA, United States*
Courtney R Buckey, *Mayo Clinic, Phoenix, AZ, United States*
Jing Cai, *The Hong Kong Polytechnic University, Hong Kong*
Ashley Cetnar, *James Cancer Hospital, Columbus, OH, United States*
Quan Chen, *University of Kentucky, Lexington, KY, United States*
Shifeng Chen, *University of Maryland, Baltimore, MD, United States*
Ting Chen, *New York University, New York, NY, United States*
Luca Cozzi, *Ospedale San Giovanni, Porto Valtravaglia, Italy*
Ryan Foster, *Atrium Health System, Concord, NC, United States*
Christopher Furweger, *The European Cyberknife Center, Germany*
Song Gao, *Health Science Center of PKU, Beijing, China*
AnneLise Giebeler, *California Protons Cancer Therapy Center, San Diego, CA, United States*
Kent Gifford, *UT MD Anderson Cancer Center, Houston, TX, United States*
Bingqi Guo, *Cleveland Clinical Learner College of Medicine, Cleveland, OH, United States*
Samantha Hedrick, *Provision Proton, Knoxville, TN, United States*
Antonio Herreros, *Hospital Clinic de Barcelona, Spain*
Yanle Hu, *Mayo Clinic, Phoenix, AZ, United States*
Donglai Huo, *University of Colorado, CO, United States*
Xun Jia, *University of Texas Southwestern Medical Center, Dallas, TX, United States*
Xiance Jin, *1st Affiliated Hospital of Wenzhou, China*
Rao Khan, *Washington University School of Medicine, St. Louis, MO, United States*
Stephen Kry, *UT MD Anderson Cancer Center, Houston, TX, United States*
Rajat Kudchadker, *UT MD Anderson Cancer Center, Houston, TX, United States*
Lalith Kumaraswamy, *Novant Health, Winston-Salem, NC, United States*
Heng Li, *Johns Hopkins, Washington, DC, United States*
Haibo Lin, *New York Proton Center, New York, NY, United States*
Liyong Lin, *Emory University, Atlanta, GA, United States*
Holly Lincoln, *Yale New Haven Hospital, Waterford, CT, United States*
Wei Liu, *Mayo Clinic Arizona, Phoenix, AZ, United States*
Wu Liu, *Stanford University, Stanford, CA, United States*
Bo Lu, *University of Florida, Gainesville, FL, United States*
Wei Luo, *University of Kentucky, Lexington, KY, United States*
Weiguo Lu, *University of Texas, Dallas, TX, United States*
Harish Malhotra, *Roswell Park Cancer Institute, Roswell Park, NY, Unites States*
Weiguo Lu, *University of Texas, Dallas, TX, United States*
Pietro Mancosu, *Humanitas Cancer Center, Porto Valtravaglia, Italy*
Quinn Matthews, *BC Cancer Agency - Centre for the North, Prince George, BC, Canada*
Geoff Nelson, *Huntsman Cancer Hospital at the University of Utah, Salt Lake City, UT, United States*
Jennifer O'Daniel, *Duke University, Duke, NC, United States*
Cheng Peng, *University of Pennsylvania, Philadelphia, PA, United States*
Matthew Podgorsak, *Roswell Park Cancer Institute, Buffalo, NY, United States*
Marija Popovic, *McGill University Health Centre, Montreal, Canada*
Richard Popple, *The University of Alabama at Birmingham, Birmingham, AL, United States*
Sharon Qi, *David Geffen School of Medicine at UCLA, Los Angeles, CA, United States*
Sarah Quirk, *Tom Baker Cancer Centre, Canada*
Prema Rassiah-Szegedi, *University of Utah, Salt Lake City, UT, United States*
Susan Richardson, *Swedish Medical Center-Tumor Institute, Seattle, WA, United States*
Yi Rong, *University of California Davis, Sacramento, CA, United States*
Narayan Sahoo, *UT MD Anderson Cancer Center, Houston, TX, United States*
Chengyu Shi, *New York Proton Center, New York City, NY, United States*
Eric Slessinger, *Slessinger Enterprises LLC, Bayside, NY, United States*
Joel St.-Aubin, *University of Iowa, Iowa City, IA, United States*
Fan-Chi F Su, *University of Utah, Salt Lake City, UT, United States*
Zhong Su, *University of Florida University of Florida, Jacksonville, FL, United States*
Baozhou Sun, *Washington University in St. Louis, St. Louis, MO, United States*
Martin W. Szegedi, *University of Utah, Salt Lake City, UT, United States*
Frank Verhaegen, *Maastricht University, Netherlands*
Jose Eduardo Villarreal-Barajas, *Royal Devon and Exeter NHS Foundation Trust, UK*
Brian Wang, *Yale University, New Haven, CT, United States*
Iris Wang, *Roswell Park Cancer Center, Roswell Park, NY, United States*
Jing Wang, *UT Southwestern Medical Center, Dallas, TX, United States*
Xiaochun Wang, *UT MD Anderson Cancer Center, Houston, TX, United States*
Ning Wen, *Henry Ford Health System, Detroit, MI, United States*
Qiuwen Wu, *Duke University, Durham, NC, United States*
Guanghua Yan, *University of Florida, Gainesville, FL, United States*
Jinzhong Yang, *UT MD Anderson Cancer Center, Houston, TX, United States*
Jun Yang, *Alliance Oncology, Drexel University, Havertown, PA United States*
Ruije Yang, *Peking University Third Hospital, Beijing, China*
Xiaofeng Yang, *Emory University School of Medicine, Atlanta, GA, United States*
Lingshu Yin, *University of Pennsylvania, Philadelphia, PA, United States*
Amy Shu-Jung Yu, *Stanford University, United States*
Grace Zeng, *Trillium Health Partners, University of Toronto, Mississauga, ON, Canada*
Geoffrey Zhang, *H. Lee Moffitt Cancer Center, Tampa, FL, United States*
Miao Zhang, *Memorial Sloan Kettering Cancer Center, New York, NY, United States*
Qinghui Zhang, *St. Vincent Medical Center, New York, NY, United States*
Xiaodong Zhang, *UT MD Anderson Cancer Center, Houston, TX, United States*
Yin Zhang, *Rutgers Cancer Institute, New Brunswick, NJ, United States*
Yongbin Zhang, *University of Cincinnati, Cincinnati , OH, United States*
Bo Zhao, *Memorial Sloan Kettering Cancer Center, Uniondale, NY, United States*
Dandan Zheng, *University of Nebraska Medical Center, Omaha, NE, United States*
Su-Min Zhou, *University of Nebraska Medical Center, Omaha, NE, United States*
Dengsong Zhu, *Saint Thomas Hospital, Murfreesboro, TN, United States*

### Associate Editors – Medical Imaging

Trevor Andrews, *Washington University St Louis, St Louis, MO, United States*
Minsong Cao, *UCLA School of Medicine, Los Angeles, CA, United States*
Idris Elbakri, *CancerCare Manitoba, Winnipeg, MB, Canada*
Frank Goerner, *Queen's Medical Center, Honolulu, HI, United States*
Dimitre Hristov, *Stanford University Cancer Center, Stanford, CA, United States*
Donglai Huo, *University of Colorado, Denver, CO, United States*
Ho-Ling Anthony Liu, *UT MD Anderson Cancer Center, Houston, TX, United States*
Xinming Liu, *UT MD Anderson Cancer Center, Houston, TX, United States*
Osama Mawlawi, *UT MD Anderson Cancer Center, Houston, TX, United States*
Kwan-Hoong Ng, *University of Malaysia, Malaysia*
Mi-Ae Park, *Harvard Medical School, United States*
John Rong, *UT MD Anderson Cancer Center, Houston, TX, United States*
Tzu-Chin Shih, *China Medical University, Beigang Township, Taiwan*
Brian Taylor, *Virginia Commonwealth University, Richmond, VA, United States*
Chang-Ying Yang, *British Columbia Cancer Agency, Kelowna, BC, Canada*
Yingli Yang, *UCLA School of Medicine, Los Angeles, CA, United States*
Jie Zhang, *University of Kentucky College of Medicine*

### Associate Editor – Radiation Measurements

Salahuddin Ahmad, *University of Oklahoma HSC, OK, United States*

### Associate Editors – Radiation Protection and Regulations

Sarah Hughes, *University of Louisville, Louisville, KY, United States*
James Rodgers, *MedStar RadAmerica Cyberknife Program, Baltimore, MD, United States*

### Associate Editors – Management and Professional Topics

Per Halvorsen, *Lahey Health and Medical Center, Burlington, MA, United States*
Douglas Pfeiffer, *Boulder Community Health, Boulder, CO, United States*

### Associate Editor – Non–Ionizing Topics

Timothy C. Zhu, *University of Pennsylvania, PA, United States*

### Associate Editor – Other Topics

Timothy D Solberg, *University of Washington, Seattle, WA, United States*

**Disclaimer**

The Publisher, American Association of Physicists in Medicine, and Editors cannot be held responsible for errors or any consequences arising from the use of information contained in this journal; the views and opinions expressed do not necessarily reflect those of the Publisher, American Association of Physicists in Medicine and Editors, neither does the publication of advertisements constitute any endorsement by the Publisher, American Association of Physicists in Medicine, and Editors of the products advertised.

**Open Access and Copyright**

All articles published in *Journal of Applied Clinical Medical Physics* are fully open access: immediately freely available to read, download and share. All articles are published under the terms of the Creative Commons Attribution License (CC-BY). The CC-BY license permits use, distribution and reproduction in any medium, provided the original work is properly cited.

**Production Editor: Reshma Razack** (email: ACMP@wiley.com).

For submission instructions and all the latest information, visit wileyonlinelibrary.com/journal/jacmp
